# Effect of pressure therapy for treatment of hypertrophic scar

**DOI:** 10.1097/MD.0000000000016263

**Published:** 2019-06-28

**Authors:** Hao Zhang, Hao-yan Wang, Da-li Wang, Xiao-dong Zhang

**Affiliations:** a Department of Plastic Burn and Cosmetic Center; b Department of Neurosurgery; c Department of Ophthalmology, First Affiliated Hospital of Jiamusi University, Jiamusi, China.

**Keywords:** effect, hypertrophic scar, pressure therapy, safety

## Abstract

**Background:**

Pressure therapy (PST) has been reported for the treatment of hypertrophic scar (HS) effectively. However, no study has assessed its effect and safety systematically. Therefore, this study will investigate its effect and safety for patients with HS.

**Methods:**

A comprehensive literature search will be performed from the electronic databases and grey literatures. The electronic databases include MEDILINE, EMBASE, Cochrane Library, Web of Science, Allied and Complementary Medicine Database, Chinese Biomedical Literature Database, and China National Knowledge Infrastructure. All of them will be searched from inception to the present without language restrictions. Any randomized controlled trials on assessing the effect and safety of PST on HS will be considered for inclusion. In addition, we will also search grey literature to avoid missing any potential studies. RevMan V.5.3 software will be utilized for statistical analysis.

**Results:**

This study will provide the most recent evidence of PST on HS by evaluating primary outcomes of scar pruritus and improvement of scar; and secondary outcomes of scar blood flow, elasticity, volume, pain and burning. In addition, we will also evaluate adverse events.

**Conclusion:**

This study will provide up-to-date evidence of PST in patients with HS.

Systematic review registration: PROSPERO CRD42019136627.

## 1 Introduction

Hypertrophic scar (HS) is a kind of visible and elevated scar, which often does not spread into surrounding tissues and regress spontaneously.^[1–3]^ It is often characterized by proliferation of the dermal tissue. This tissue often accompanies excessive deposition of fibroblast-derived extracellular matrix proteins and especially collagen, which lasts long term with persistent inflammation and fibrosis.^[4–6]^

A variety of managements are reported to treat this disorder.^[7–20]^ These managements include small-wave incision, intralesional interferon, streptomycin/lignocaine local infiltration, topical and intralesional corticosteroids, intralesional bleomycin, laser therapy, silicone gel, and pressure therapy (PST).^[7–20]^ However, up to date, the optimal treatment method has not been established. Although several clinical studies have reported that PST can treat HS effectively,^[21–25]^ no systematic review has been conducted to assess its effect and safety in patients with HS. Thus, in this study, we will systematically investigate its effect and safety in patients with HS.

## 2 Methods

### 2.1 Ethics and dissemination

This study does not require ethic approval because we will not analyze individual patient data. This study will be published on peer-reviewed journals.

### 2.2 Registration

This study has been registered in the PROSPERO with number of CRD42019136627. We will report this study in accordance with the guideline of Preferred Reporting Items for Systematic Reviews and Meta-Analysis (PRISMA) Protocol statement.^[26]^

### 2.3 Eligibility criteria

#### 2.3.1 Types of studies

All randomized controlled trials (RCTs) assessing the effect and safety of PST in patients with HS will be included. However, we will exclude non-clinical studies, non-RCTs, and quasi-RCTs.

#### 2.3.2 Types of participants

Participants with HS will be included in this study regardless their race, age, gender, and economic status.

#### 2.3.3 Types of treatments

Experimental group: patients receive PST for HS.

Control group: patients receive any treatments except HS.

#### 2.3.4 Types of outcome measurements

Primary outcomes include scar pruritus, as measured by 4-item Itch Questionnaire or any other associated scales; and improvement of scar, as assessed by photography or relevant tools.

Secondary outcomes consist of clinic measurements of scar blood flow, elasticity, and volume; and patients’ subjective complaints of pain and burning; as well as adverse events.

### 2.4 Data sources and search strategy

We will perform a comprehensive literature search from the electronic databases of MEDILINE, EMBASE, Cochrane Library, Web of Science, Allied and Complementary Medicine Database, Chinese Biomedical Literature Database, and China National Knowledge Infrastructure from inception to the present without language restrictions. The comprehensive search strategy for MEDLINE is presented in Table 1. Similar search strategy will be adapted to any other electronic databases. Additionally, we will also search grey literature, such as dissertations, clinical registry, and reference lists of relevant reviews.

**Table 1:**
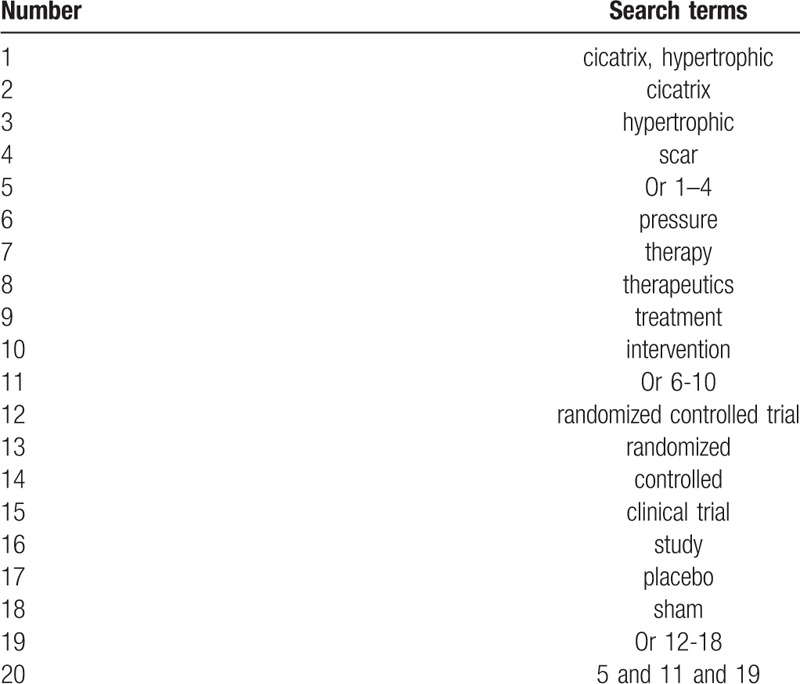
Search strategy for MEDLINE.

### 2.5 Data collection and management

#### 2.5.1 Study selection

Two reviewers will independently perform study selection according to the predefined eligibility criteria. Any different opinions regarding the study selection between 2 reviewers will be settled down by a third reviewer through discussion. The whole process will consist of 2 steps. First, all searched literature will be scanned by titles and abstracts, and all irrelevant studies will be excluded. Second, all remaining literature will be read by full-texts and will be further judged if they meet final eligibility criteria. We will present the results of study selection in PRISMA flow diagram.

#### 2.5.2 Data extraction

Two reviewers will independently perform data extraction in accordance with predefined data extraction sheet. A third reviewer will help to solve any disagreements between 2 reviewers. The extracted information includes study information (such as title, author, region, etc), patient characteristics (such as age, sex, diagnostic criteria, etc), study methods (such as sample size, randomization, concealment, blinding, etc), treatment methods (such as intervention name, dosage, frequency, duration, etc), and outcome measurements (such as primary and secondary outcomes, and safety).

#### 2.5.3 Missing data management

If there is insufficient or missing data, we will contact primary corresponding author via email to request that. If that data will not be achievable, only available data will be analyzed. In addition, the potential impacts of missing data will be discussed.

### 2.6 Methodological quality assessment

Cochrane risk of bias tool will be used to assess the methodological quality for each eligible study. Two reviewers will independently assess the methodological quality for all eligible studies. Any disagreements regarding the methodological quality assessment will be worked out by a third reviewer through discussion. This tool comprises of 7 domains, and each one is further judged as high, unclear, and low risk of bias.

### 2.7 Statistical analysis

RevMan V.5.3 software will be used to operate statistical analysis. The risk ratio and 95% confidence intervals (CIs) will be utilized to express dichotomous data. A weighted mean difference or a standard mean difference with 95% CIs will be employed to present continuous outcomes.

We will use *I*^*2*^ statistic test to check heterogeneity among eligible studies. If the value of *I*^*2*^ is less than 50%, reasonable heterogeneity is identified, and a fixed-effect model will be used to pool the data. Otherwise, if the value of *I*^*2*^ is more than 50%, significant heterogeneity is identified, and a random-effect model will be used to pool the data. Additionally, we will also perform subgroup analysis to explore any possible factors for substantial heterogeneity. We will carry out meta-analysis if heterogeneity is reasonable. If there is still substantial heterogeneity after subgroup analysis, we will not perform data pooling and meta-analysis. Instead, we will report narrative summary.

Subgroup analysis will be conducted based on the different study, interventions, controls, and outcomes. In addition, we will carry out sensitivity analysis to check the stability and robustness of outcome results by removing low quality studies. Furthermore, funnel plots and Egger linear regression test will also be carried out if more than 10 eligible studies are entered in this study.

## 3 Discussion

This study will investigate the effect and safety of PST in patients with HS systematically. A comprehensive literature search will be performed without any language restrictions. We will also retrieve grey literature to avoid missing any potential eligible studies. Two reviewers will independently carry study selection, data extraction, and methodological quality assessment. Any divergences between 2 reviewers will be solved by a third reviewer through discussion. The findings of this study will summarize the latest evidence on assessing the effect and safety of PST in patients with HS. It may benefit either the future research or the clinical practice.

## Author contributions

**Conceptualization:** Hao Zhang, Da-li Wang, Xiao-dong Zhang.

**Data curation:** Hao Zhang, Hao-yan Wang, Xiao-dong Zhang.

**Formal analysis:** Hao-yan Wang, Da-li Wang, Xiao-dong Zhang.

**Funding acquisition:** Hao Zhang.

**Investigation:** Hao Zhang.

**Methodology:** Hao-yan Wang, Da-li Wang, Xiao-dong Zhang.

**Project administration:** Hao Zhang.

**Resources:** Hao-yan Wang, Da-li Wang, Xiao-dong Zhang.

**Software:** Hao-yan Wang, Da-li Wang, Xiao-dong Zhang.

**Supervision:** Hao Zhang.

**Validation:** Hao Zhang, Da-li Wang, Xiao-dong Zhang.

**Visualization:** Hao Zhang, Hao-yan Wang, Xiao-dong Zhang.

**Writing – original draft:** Hao Zhang, Hao-yan Wang, Da-li Wang.

**Writing – review & editing:** Hao Zhang, Hao-yan Wang, Da-li Wang, Xiao-dong Zhang.
